# Supplementary material for: The genome sequence of the White-speck moth, Mythimna unipuncta (Haworth, 1809) (Lepidoptera: Noctuidae)
Source: Wellcome Open Res. 2026 Sep 11;11:623. [Version 1] doi: 10.12688/wellcomeopenres.27767.1 (PMC13684022; doi:10.12688/wellcomeopenres.27767.1)
Supplement: Peer review history

# Reviewer report: Reviewer response for version 1

**DOI:** 10.21956/wellcomeopenres.30558.r170697

**Published:** 2026-10-20

**Authors:** Alvaro G. Hernandez

**Affiliations:** 1 University of Illinois Urbana-Champaign, Urbana, Illinois, USA

Summary

 The authors present a Hi-C phased, chromosome-level genome assembly of the White-speck moth, Mythimna unipuncta (Noctuidae), generated within Project Psyche using PacBio Revio HiFi, and Hi-C data. Haplotype 1, which was proposed as the reference assembly over haplotype 2, spans 758.97 Mb, with 99.67% assigned to 30 autosomes and the Z and W chromosomes. It has a QV of 66.2 and a BUSCO completeness of 98.8%. Ensembl annotated 14,675 protein-coding genes. The assembly is of high quality and meets the Earth BioGenome Project standards. My comments are mostly requests for clarification and additional methodological details, and I recommend approval after minor revisions.

 Review points

 The Femto Pulse system is first mentioned on page 4 under Nucleic acid extraction, but its full name and manufacturer (Agilent Femto Pulse Automated Pulsed Field CE Instrument, Agilent Technologies) are given on page 5. I suggest defining it at first use. The same applies to the Qubit on pages 4 and 5.

 The PacBio sequencing description is unclear. A single library (PSYCHE15123103) is described, but the text then mentions that the prepared libraries selected for multiplexing were pooled based on genome size, library molarity and the intended plex level, and that they were sequenced in a 2-plex run. Clarify whether more than one library was prepared, or whether this library was multiplexed with a library from a different project.

 Provide the SMRT Link software version (page 5).

 State how many Hi-C libraries were prepared from the head sample, which NovaSeq X flow cell type was used (1.5B, 10B or 25B), and whether full or partial lanes were used. The authors mention that 333.27 million reads were obtained, but not which flow cell or lanes were used. Also, page 6 says 333.27 million reads, whereas Table 1 says 333.27 million read pairs.

 Page 5: What filtering method was used before generating the k-mer counts?

 Decontamination was done using the ASCC pipeline. Were any symbionts, cobionts or other organisms of interest detected? If so, it would be worth mentioning them.

 I suggest reporting the repeat content of the assembly itself, in addition to the GenomeScope estimate.

 The average transcript length of 20,430.69 bp appears to correspond to the genomic span of the transcripts, including introns, rather than the length of the spliced mRNA, which should be a few kilobases. I suggest clarifying which measure is reported, and consider also reporting the average spliced transcript length and the average coding sequence length, as provided in the Ensembl statistics.

 What evidence was used for the annotation? Was RNA-Seq or protein data used?

 The Iberfauna, 2008 reference cited on page 3 does not appear in the reference list on page 17.

 Rhie et al.. (2021) on page 14 has a double period.

 In Table 4, # 40 and # 90% appear to be errors for the greater than (>) symbol.

Are the datasets clearly presented in a usable and accessible format, and the assembly and annotation available in an appropriate subject-specific repository?

Yes

Are sufficient details of the sequencing and extraction, software used, and materials provided to allow replication by others?

Yes

Are the rationale for sequencing the genome and the species significance clearly described?

Yes

Are the protocols appropriate and is the work technically sound?

Yes

Reviewer Expertise:

DNA Sequencing technologies and applications.

I confirm that I have read this submission and believe that I have an appropriate level of expertise to confirm that it is of an acceptable scientific standard.

# Reviewer report: Reviewer response for version 1

**DOI:** 10.21956/wellcomeopenres.30558.r170692

**Published:** 2026-10-13

**Authors:** Michael Goodisman, Michael Catto

**Affiliations:** 1 Georgia Institute of Technology, Atlanta, USA

Meier and Blaxter present the construction and annotation of the genome of the White-speck moth (Mythimna unipuncta, Haworth). The assembly is comprised of two haplotypes, which differ in their characteristics: haplotype 1 (758.97 Mb) was assembled to chromosome level, covering 99.67% of the sequence, while haplotype 2 (683.60 Mb) remains at scaffold level. The mitochondrial genome was also assembled and has a length of 15.38 kb. Overall, this work provides a valuable genomic resource for an agricultural pest species, but there are some items we think should be addressed to help improve the quality of this resource.

 Comments

 •    We don’t fully understand how it is possible to distinguish the ‘haplotypes’. That is, the research team started with a diploid individual which has 32 chromosomes. But how do the methods appropriately assign each pair of chromosomes into a distinct haplotype?  What does this mean? Did the research team have access to the parental genotypes, which might aid in distinguishing haplotypes?  One can understand the idea of a haplotype when considering sequences on a single chromosome. Or if genetic information is a haploid phase, such as a gamete. But how is a multi-chromosome haplotype distinguished in a single diploid individual?

 •    ‘Haplotype 1’ was assembled to chromosome level, whereas ‘haplotype 2’ was assembled only to scaffold level. Could the authors clarify the reason for this difference (e.g., coverage, Hi-C contact density, or phasing difficulties)? A fully phased, chromosome-level assembly of both haplotypes would greatly facilitate comparisons between the two parental lineages.

 •    Could the authors expand their discussion of how heterozygosity may have affected the gene annotation? In particular, could high heterozygosity between haplotypes have led to redundant gene models being annotated as separate paralogs rather than allelic variants? Please clarify whether the annotation pipeline distinguishes between allelic gene variants (the same gene on the maternal vs. paternal chromosomes) and true gene duplications. Without such a distinction, gene counts may be inflated, which would affect downstream comparative analyses.

 •    Were any metrics used to assess the quality of the genome annotation, such as a BUSCO completeness score for gene space, as was done for the assembly itself?

 •    How does the predicted gene count compare to those of related Mythimna species, and is it consistent with expectations for Lepidoptera generally?

 •    Was the assembled mitochondrial genome deposited in a public database (e.g., NCBI GenBank), and if so, could the accession number be provided?

 •    Figure 1 shows a reasonable photo of the adult stage of the moth.  But, given its agricultural importance, it would be nice if the organismal photo could also include the larval stages as well.

Are the datasets clearly presented in a usable and accessible format, and the assembly and annotation available in an appropriate subject-specific repository?

Yes

Are sufficient details of the sequencing and extraction, software used, and materials provided to allow replication by others?

Yes

Are the rationale for sequencing the genome and the species significance clearly described?

Yes

Are the protocols appropriate and is the work technically sound?

Yes

Reviewer Expertise:

Genetics

We confirm that we have read this submission and believe that we have an appropriate level of expertise to confirm that it is of an acceptable scientific standard, however we have significant reservations, as outlined above.

# Reviewer report: Reviewer response for version 1

**Published:** 2026-09-11

**Authors:** Automated Benchmarking

**Affiliations:** 1 Wellcome Sanger Institute, Hinxton, UK

As this article is a Genome Note, Automated Benchmarking has been used to determine whether the reported assembly passes three defined checks established by the Earth BioGenome Project: the raw and assembled data are publicly available, the Earth BioGenome Project three-part metric is met, and the BUSCO score passes the stated threshold. For Mythimna unipuncta (ilMytUnip1), all three checks passed. Data available: pass. ENA study PRJEB82302, raw-read accessions ERR13946512 and ERR13947541, and assembly accessions GCA_964503205.1 and GCA_964504095.1 are public. Earth BioGenome Project three-part metric: pass. Haplotype 1 is reported as 6.C.Q66, meeting the 6.C.Q40 reference standard. BUSCO: pass. Haplotype 1, which is proposed as the reference assembly for this species, has 98.8% complete BUSCOs (98.3% single-copy and 0.5% duplicated). It meets the target of greater than 90% complete and single-copy BUSCOs. This decision uses values reported in the Genome Note and public accession records.

Reviewer Expertise:

NA

I confirm that I have read this submission and believe that I have an appropriate level of expertise to confirm that it is of an acceptable scientific standard.
